# Elongatoolithid eggs containing oviraptorid (Theropoda, Oviraptorosauria) embryos from the Upper Cretaceous of Southern China

**DOI:** 10.1186/s12862-016-0633-0

**Published:** 2016-03-25

**Authors:** Shuo Wang, Shukang Zhang, Corwin Sullivan, Xing Xu

**Affiliations:** College of Life Sciences, Capital Normal University, Beijing, 100048 China; Key Laboratory of Vertebrate Evolution and Human Origins of Chinese Academy of Sciences, Institute of Vertebrate Paleontology and Paleoanthropology, Chinese Academy of Sciences, Beijing, 100044 China

**Keywords:** Development, Theropod dinosaur, Histology, Ontogenetically variable features, Oviraptorosaur, Pathology

## Abstract

**Background:**

Oviraptorids, like many other dinosaurs, clearly had a complex pattern of skeletal growth involving numerous morphological changes. However, many ontogenetic skeletal changes in oviraptorids were previously unclear due to the lack of well preserved specimens that represent very young developmental stages.

**Results:**

Here we report three elongatoolithid dinosaur eggs from the Upper Cretaceous Nanxiong Formation of Nankang District, Ganzhou City, Jiangxi Province, China that contain *in ovo* embryonic skeletons. The eggs themselves show diagnostic features of the oofamily Elongatoolithidae, whereas the embryos are identified as taxonomically indeterminate oviraptorids. The three new specimens display pathological eggshell features, including double-layered and multilayered cones in the columnar layer, which probably result from high levels of pathogenic trace elements in the environment. Nevertheless, the skeletons of the preserved embryos exhibit no structural or histological abnormalities. Comparisons between the new embryos and other oviraptorid specimens reveal 20 osteological features that appear to change substantially during ontogeny in oviraptorids. For example, the dorsoventral height of the skull increases more rapidly than the anteroposterior length during oviraptorid ontogeny, and the initially paired nasals fuse at an early stage, presumably facilitating growth of a crest.

**Conclusions:**

The new specimens represent the first known oviraptorid embryos associated with pathological eggshells. The absence of structural and histological abnormalities indicates the environmental factor that led to the eggshell pathologies did not affect the skeletal development of the oviraptorids themselves. As in tyrannosaurids, but in contrast to the situation in other maniraptorans, the oviraptorid skull becomes proportionally dorsoventrally deeper during ontogeny. Although oviraptorids and therizinosauroids occupy broadly the same grade of maniraptoran evolution, the embryonic ossification patterns of the vertebral column and furcular hypocleidium appear to differ significantly between the two clades. The limb proportions of juvenile oviraptorids indicate that they were bipedal, like adults. Oviraptorids may have differed greatly from therizinosauroids in their growth trajectories and locomotor modes during early post-hatching ontogeny, essentially occupying a different ecological niche.

## Background

Non-avian dinosaurs, and particularly theropods, have attracted considerable research interest in recent years due to their status as close relatives of birds [1–6]. However, scientific attention has not been distributed equally across all aspects of dinosaur biology and evolution. Most studies have focused on describing new taxa, analyzing their interrelationships and reconstructing their evolutionary history [7–9], and even the relatively small number dealing with the ontogenetic development of the skeleton have primarily concentrated on late juvenile to adult stages [6, 10–13].

Enough ontogenetic information has been acquired from embryonic specimens to not only demonstrate the existence of developmental disparities among different non-avian dinosaurs, but also help to clarify the potential of ontogenetically variable characters to confound phylogenetic analyses of theropods. Several embryonic theropods have been reported in the last two decades, coming from all over the world and representing a variety of taxa including the basal tetanuran *Torvosaurus* [14], the basal coelurosaur *Lourinhanosaurus* [15–17], the maniraptoran *Troodon* [18], and taxonomically indeterminate members of the clades Therizinosauroidea [19, 20] and Oviraptorosauria [21–24].

Oviraptorids are a group of oviraptorosaurian theropod dinosaurs that are currently known only from the Upper Cretaceous deposits of the Mongolian plateau and central and southern China [25–28]. A few embryonic oviraptorid specimens have been reported from these regions. Norell et al. [21, 22] described an oviraptorid embryo MPC-D100/971 from the Upper Cretaceous Djadokhta Formation of Ukhaa Tolgod, Mongolia, and concluded that oviraptorids must have generally resembled precocial birds in their prenatal ontogeny. Weishampel et al. [23] described three other oviraptorid embryos from a single nest at Bugin-Tsav, Mongolia, documenting several ontogenetically variable features and proposing that asynchronous egglaying must have been present in oviraptorids [23]. Cheng et al. [24] briefly reported two embryos from the Upper Cretaceous Nanxiong Formation of Ganzhou City, Jiangxi Province, southern China, and assigned them to the oviraptorid taxon *Heyuannia huangi* without discussing patterns of morphological change during ontogeny in this species [24].

Here, we describe three newly collected elongatoolithid eggs from Nankang District in Ganzhou City, each of which contains a nearly complete *in ovo* oviraptorid skeleton. These specimens preserve the best currently available examples of several parts of the skeleton that were previously poorly known in oviraptorid embryos, including the cranium, vertebral column and pelvis, and therefore contribute significantly to our emerging understanding of oviraptorid ontogeny.

## Methods

### Institutional abbreviations

CM, Chimei Museum, Tainan, China; GIN, Geological Institute of the Mongolian Academy of Science, Ulaan Baatar, Mongolia; HGM, Henan Geological Museum, Zhengzhou, China; IGM, Institute of Geology, Ulaan Baatar, Mongolia; IVPP, Institute of Vertebrate Paleontology and Paleoanthropology, Chinese Academy of Sciences, Beijing, China; MPC, Mongolian Paleontological Center, Ulaan Baatar, Mongolia; NMNS, National Museum of Natural Science, Taipei, China; ZPAL, Institute of Palaeobiology of the Polish Academy of Sciences, Warsaw, Poland.

### Source of the embryos and geological context

**Fig. 1:**
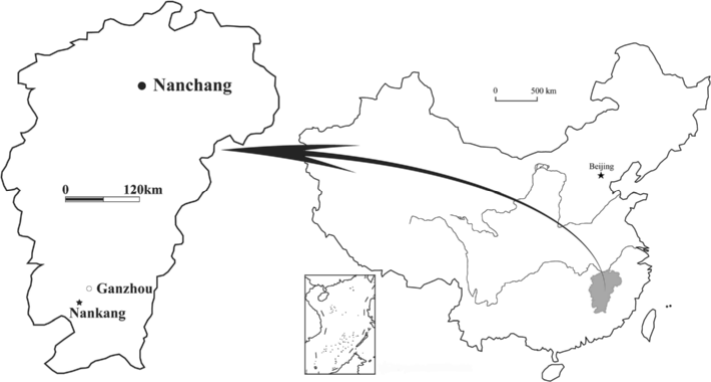
Map showing the region that produced the elongatoolithid eggs with *in ovo* embryos IVPP V20182, IVPP V20183 and IVPP V20184

The eggs with *in ovo* oviraptorid embryos described in this paper were obtained from a local farmer by Huachun Shan, an entrepreneur who is committed to keeping scientifically important fossils within the public trust. She donated these specimens to the IVPP, and they were subsequently accessioned as IVPP V20182 ~ V20184 (note that the specimens are freely available for study by qualified researchers). The only information concerning the source of these embryonic specimens provided by the farmer was that they were collected from red beds somewhere in Nankang District, Ganzhou City, Jiangxi Province (Fig. 1), and did not come from the same clutch or even the same locality. The red beds exposed in Nankang District belong to the Upper Cretaceous Nanxiong Group, which has yielded a variety of fossils including the turtle *Nanhsiungchelys* [29]; the squamate *Chianghsia nankangensis* [30]; the titanosauriform *Gannansaurus sinensis* [31]; the titanosaurian *Titanosaurus* sp. [29]; the tyrannnosaurid *Qianzhousaurus sinensis* [32]; the basal oviraptorosaur *Nankangia jiangxiensis* [33]; the oviraptorids *Banji long* [27], *Ganzhousaurus nankangensis* [34] and *Jiangxisaurus ganzhouensis* [35]; isolated theropod teeth [36]; fossil eggs assigned to the oospecies *Paraspheroolithus irenensis*, *Ovaloolithus chinkangkouensis*, *Macroolithus rugustus* and *Macroolithus yaotunensis* [37–39]; and the gastropods *Truncatella maxima* and *Rubeyella carinata* [29], which indicate that the Nanxiong Group was deposited toward the end of the Late Cretaceous (Campanian-Maastrichtian) [27, 29].

### CT scanning

The eggs with *in ovo* oviraptorid embryos described in this paper were scanned using the industrial computerized tomography (mi-CT 450ICT, developed by the Institute of High Energy Physics, Chinese Academy of Sciences) facility at the Key Laboratory of Vertebrate Evolution and Human Origins, Institute of Vertebrate Paleontology and Paleoanthropology, Chinese Academy of Sciences. The samples were placed approximately 50 cm from the X-ray source and 40 cm from the detector. The resolution of the CT images is 160 μm, and a total of 1536 transmission images each consisting of 2048*2048 pixels were captured for each sample. The transmission images were processed using two-dimensional reconstruction software developed by the Institute of High Energy Physics, Chinese Academy of Sciences.

### Preparation and photography

**Fig. 2:**
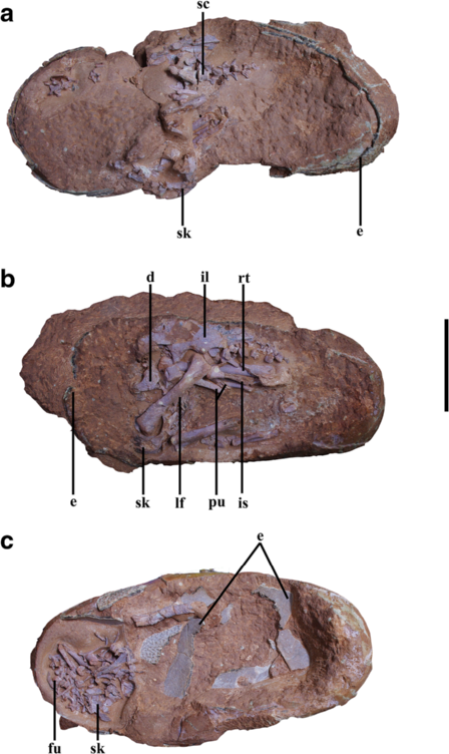
Elongatoolithid eggs from the Ganzhou area with *in ovo* embryonic skeletons. **a**, IVPP V20182; **b**, IVPP V20183; **c**, IVPP V20184; scale bar = 5 cm. Abbreviations: d, dentary; e, eggshell; fu, furcula; il, ilium; is, ischium; lf, left femur; pu, pubis; rt, right tibia; sc, sacral vertebra; sk, skull

**Fig. 3:**
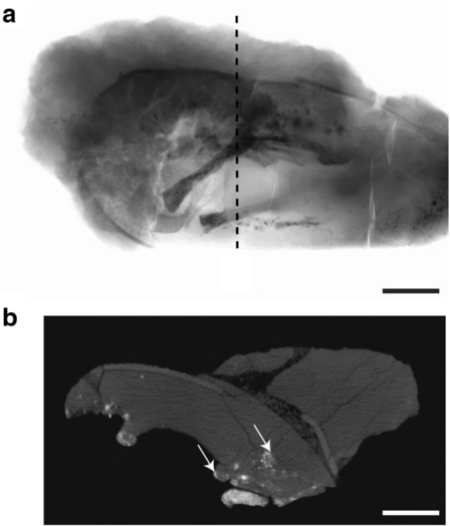
CT images of IVPP V20183. **a**, volumetric model generated from superimposed CT slices, with a black dashed line marking the position of the slice shown in B; scale bar = 5 cm; **b**, selected CT slice showing the limited density contrast between the bone and matrix (white arrows mark the bones); scale bar = 1 cm

The eggs were nearly complete when collected, and the embryonic skeletons were prepared at the IVPP (Fig. 2). The matrix inside the eggs is identical in color to that surrounding the eggs, and CT scanning of IVPP V20183 (Fig. 3a) and the other two specimens did not reveal any discontinuities that might represent boundaries between blocks that had been artificially combined. As a result, we are completely confident that the association between the eggs and the embryos is entirely natural. Matrix was removed with hand tools, and the bone was consolidated as necessary with cyanoacrylate adhesive (Aibida Company). Because CT-scanning failed to distinguish clearly between matrix and bone (Fig. 3b), the skeletal descriptions presented below are primarily based on visual observation of all available bones under a stereo microscope (Olympus SZX12). The specimens were photographed primarily with a Pentax smc DAL digital camera, although an Olympus DP70 Digital Camera System was used to obtain digital images of some tiny structures through the stereo microscope. Detailed images of eggshell and bone histological slides were obtained with a Leica MPS 60 Camera System through a transmitted light microscope (Leica DMRX).

### Histological methods

Eggshell samples from all three specimens were examined. Histological bone samples were obtained by removing 5-mm-long mid-diaphyseal portions of the tibiae of IVPP V20182 and IVPP V20183, but no comparable histological section of IVPP V20184 is available because the tibiae were not preserved in this specimen. For thin sectioning, samples were first gently cleaned with airflow and then embedded under vacuum in blocks of acrylic resin (Technovit 7200 VLC). Each block was cut, transverse to the bone shaft, using an EXAKT 300CP automatic microtome with a 0.35 mm wide blade (EXAKT-300CL, EXAKT Band System). The cut surface of each block was polished using an EXAKT 400CP variable speed grinder-polisher with P500 and P4000 polishing paper successively. Acrylic resin was then applied to the polished surface of the block under vacuum. After the resin filled any pores, this surface was glued to a glass slide using acrylic resin. Once mounted on glass, the block was cut down to an appropriate thickness (usually 120 ~ 150 μm), and the cut sections were further polished with P500 and P4000 polishing paper until their thickness had been reduced to a final value of 40–60 μm, facilitating examination by transmitted and polarized light microscopy (Leica DMRX). We used the computer program Mimics (version 10.01) to measure the percentage of porous space within the bone cortex, as a proxy for the percentage of the cortical area occupied by vascular canals [40].

## Results

### Eggs

Although some minor structural variation occurs within and among the newly collected eggs examined in this study, they share the following combination of taxonomically significant features: elongate proportions, with lengths ranging from 163.5 to 198.3 mm and widths ranging from 74.8 to 92.1 mm (Table 1); eggshell composed of cone and columnar layers separated by a clear boundary; and undulating growth lines in columnar layer lying parallel to outer surface of eggshell and visible in radial sections. These features conform to the diagnosis for Elongatoolithidae in most respects [37, 41], and the newly collected eggs are therefore referable to that oofamily. The following detailed description of eggshell is based on IVPP V20184, but generally applies to the other eggs unless otherwise specified.

**Table 1:** Selected measurements of oviraptorid eggs and embryos (mm)

Measurements	IVPP V20182	IVPP V20183	IVPP V20184
Egg size (length × width)	198.3 × 88.0	163.5 × 74.8	179.5 × 92.1
Maximum skull length	46.8		
Maximum skull height	29.2		
Maximum mandible length	38.7		
Maximum mandible height	15.2		
External naris length	6.8		
External naris height	4.5		
Lacrimal height	15.6		
Ilium length		45.5	
Preacetabular process length		18.7	
Postacetabular process length		10.6	
Acetabulum length		11.8	
Maximum femoral length		49.2 (L)	
Maximum tibial length		56.2^a^ (R)	
Maximum tibial diameter (midshaft)	4.3	4.9	
Tibial marrow cavity diameter	2.2	1.8	

^a^All estimates, due to incomplete preservation

**Fig. 4:**
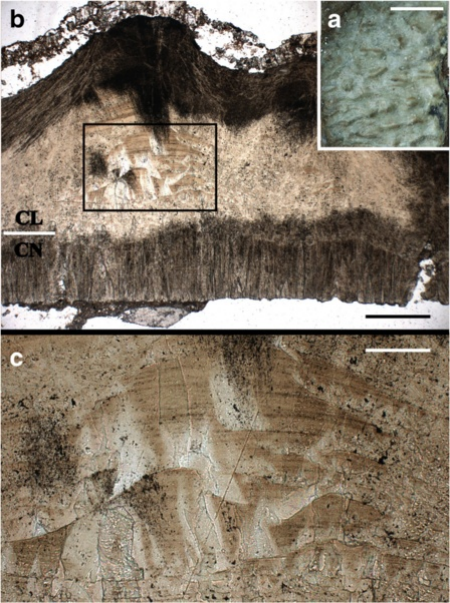
Eggshell of IVPP V20184. **a**, outer surface of the eggshell, showing the pattern of the external ornamentation; scale bar = 5 mm; **b**, radial thin section of the eggshell as seen through a light microscope, showing external ornamentation and two structural layers comprising the shell; the black box encloses pathological multilayered cones, and the horizontal white bar at left indicates boundary between layers; scale bar = 0.25 mm; **c**, close-up photograph of pathological multilayered cones shown in **b**; scale bar = 0.07 mm. Abbreviations: CL, columnar layer; CN, cone layer

The external ornamentation on the egg comprises elongate, occasionally bifurcating ridges aligned with the long axis of the egg (Fig. 4a). The eggshell thickness varies from 1.20 ~ 1.76 mm, depending on which part of the eggshell is sampled and on the height of the ornamentation at the point where the measurement is taken. The average thickness is significantly greater than in typical *Elongatoolithus* (in which this parameter is usually less than 1.20 mm), but similar to that seen in *Macroolithus* [37]. Radial thin sections of eggshell viewed microscopically under normal and polarized light do not reveal serious diagenetic alteration. The cone layer varies in thickness from 0.24 ~ 0.44 mm, and consists of narrow, closely packed, elongate cones with an average diameter of 0.12 mm. In the process of eggshell formation, these calcite crystals would have originated from nucleation sites at the base of the cone layer and grown outward until truncated by contact with the neighboring cones (Fig. 4b). Although the bases of the cones are typically intact, some cones are poorly preserved, probably due to weathering. The average thickness of the overlying columnar layer is 1.12 mm, and the ratio of the thickness of the cone layer to that of the columnar layer varies from approximately 0.25 ~ 0.30 (Table 2). The boundary between the columnar layer and the cone layer is undulant. Interestingly, double-layered and multilayered cones, structures that are considered pathological (see Discussion) [42–45], are visible in the columnar layer (Fig. 4c). Under polarized light, an extinction pattern is visible within the columnar layer, but only beneath the ridges on the outer surface of the eggshell.

**Table 2:** Selected measurements of oviraptorid eggs from China and Mongolia (mm)

	IVPP V20184	NMNS-0015726-F02-embryo-01	IVPP V9608	IGM 100/971	IGM 100/979	MPC-D100/1017	MPC-D107/15
Locality	Ganzhou, Jiangxi, China	Ganzhou, Jiangxi, China	Bayan Mandahu, China	Ukhaa Tolgod, Mongolia	Ukhaa Tolgod, Mongolia	Bugin-Tsav, Mongolia	Northern Sayr, Mongolia
Egg size	179.5 × 92.1	173.5 × 76.3	150 × 55	120 × 60^a^	180 × 65	-	140–160 × 50–60
Eggshell thickness	1.20–1.76	1.6	0.75	0.5–0.95		0.73–1.00	1.0–1.2
Cone layer thickness (C1)	0.24–0.44	0.22–0.39	0.2			0.19–0.33	
Columnar layer thickness (C2)	0.96–1.32	0.76–1.09	0.55			0.48	
C1/C2	1:3–1:4	1:3	1:2.7	1:2.7^a^		1:3–1:2	
Reference	This paper	[24]	[100]	[21]	[101]	[23]	[49]

^a^All estimates, due to incomplete preservation

Within Elongatoolithidae, the size, thickness, external ornamentation and cone-to-columnar thickness ratio of the eggs IVPP V20182 ~ V20184, as well as the presence in these eggs of an undulant boundary between the cone and columnar layers, are all features shared with some specimens that have been previously assigned to the oospecies *Macroolithus yaotunensis* [37]. However, the significance of these similarities is difficult to evaluate because *M. yaotunensis* has been used to accommodate a morphologically wide range of fossil eggs and stands in need of revision [46]. At present, we refrain from assigning IVPP V20182 ~ V20184 to any taxon below the level of Elongatoolithidae, while noting their similarity to morphotypes that have been referred to *M. yaotunensis*.

Among previously described elongatoolithid eggs associated with oviraptorosaur skeletal material, Cheng et al. [24] previously assigned the Ganzhou specimens NMNS-0015726-F02-embryo-01 and CM-41 to *Macroolithus yaotunensis* based on their overall size and microstructure, whereas Norell et al. [21] referred the egg IGM 100/971 to Elongatoolithidae indet. The eggshell of IGM 100/971 is much thinner than that of eggs that have been assigned to *Macroolithus yaotunensis*, and the cone-to-columnar thickness ratio is slightly smaller in the former specimen (Table 2). These comparisons, combined with the size of the egg and the slender ridges on the outer surface of the shell, indicate that IGM 100/971 is probably most closely related to *Elongatoolithus elongatus* among currently known oospecies [37, 47]. Although the exact size of the Bugin-Tsav oviraptorid egg MPC-D100/1017 is unknown, its eggshell thickness is similar to that of IGM 100/971 [23]. Together with the bifurcating ridges on the egg’s outer surface, the poorly defined boundary between the cone and columnar layers, and the relatively broad cones (~0.25 mm in diameter), this feature indicates that MPC-D100/1017 is similar to *Elongatoolithus* as well. The eggs beneath the ‘brooding’ oviraptorid skeleton IGM 100/797 closely resemble the newly collected specimens in their relatively large size [48], but their microstructure remains unclear. Meaningful comparisons are not possible with the eggs of *Nemegtomaia* preserved with MPC-D107/15, which have been extensively altered by diagenesis [49].

### Embryos

All three of the embryos preserved with the Ganzhou eggs lie *in ovo*. Whether these embryos represent a single taxon is uncertain, partly because the degree of stratigraphic and geographic proximity among the three specimens is unclear but also because few skeletal elements are well-preserved in more than one of them. We therefore describe these specimens separately below.

### IVPP V20182 (Fig. 5)

**Fig. 5:**
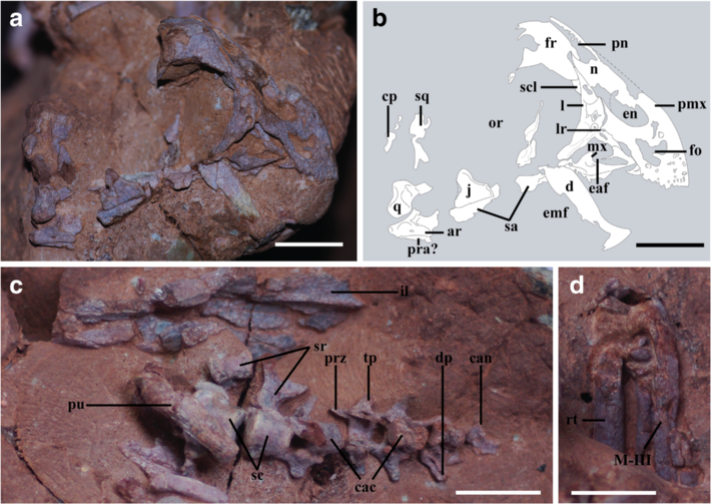
Photographs of IVPP V20182. **a**, magnified right lateral view of the nearly complete skull; scale bar = 1 cm; **b**, schematic drawing of the skull; scale bar = 1 cm; **c**, magnified ventral view of the pelvic girdle, the sacral vertebrae and the proximal caudal vertebrae; scale bar = 1 cm; **d**, magnified dorsal view of the proximal half of the metatarsals, scale bar = 1 cm. Abbreviations: ar, articular; cac, caudal centrum; can, caudal neural arch; cp, squamosal caudal process; d, dentary; dp, depression; e, external naris; eaf, external antorbital fenestra; emf, external mandibular fenestra; fo, foramen; fr, frontal; il, ilium; j, jugal; l, lacrimal; lr, lacrimal recess; mx, maxilla; n, nasal; or, orbit; pmx, premaxilla; pra, prearticular; prz, prezygapophysis; pu, pubis; q, quadrate; rt, right tibia; sa, surangular; sc, sacral centrum; scl, sclerotic plates; sr, sacral rib; sq, squamosal; tp, transverse process; MIII, metatarsal III

#### Histological description

The tibial cross section shows that the cortex is formed entirely of fibrolamellar bone that encircles the medullary cavity (Figs. 8a_1_, a_2_), as in all known embryonic dinosaurs [40]. The cross section is nearly circular, with a relatively large marrow cavity accounting for approximately half the total diameter of the bone shaft (Table 1). The fibrolamellar bone contains numerous elongate to circular vascular canals that are radial in orientation, with an overall porosity of 25.8 %. Some osteocyte lacunae are organized around the vascular canals, forming incipient primary osteons.

#### Skeletal description

This specimen preserves the most spectacular articulated cranium and mandible to have ever been reported in an embryonic oviraptorid, including examples of the premaxilla, maxilla, nasal, frontal, lacrimal, jugal, quadrate, dentary, surangular and articular in addition to a possible prearticular (Figs. 5a, b). Most of these bones are from the right side of the skull and lower jaw. Preserved postcranial skeletal elements include the posterior sacral and proximal caudal vertebrae, the partial left ilium, and partial hindlimbs. Unlike in IGM 100/971 and IVPP V20183, the rostral margin of the skull is in contact with the eggshell, indicating that the skull has probably undergone postmortem displacement (Fig. 2a).

The overall shape of the skull is sub-trapezoidal in lateral view, as is typical in all juvenile and adult oviraptorids (Fig. 5b) [23, 27, 28, 50]. The maximum length of the preserved skull (from the rostral margin of the premaxilla to the posterior end of the paroccipital process, Table 1) is around 46.8 mm, whereas the maximum height (from the dorsal surface of the frontal to the articular end of the quadrate) is about 29.2 mm.

The paired edentulous premaxillae form a beak with crenulated margins as in other oviraptorids (Figs. 5a, b) [50]. It is clear that the premaxillae remain unfused along the midline, reminiscent of their counterparts in *Incisivosaurus gauthieri*, *Caudipteryx zoui*, *Conchoraptor gracilis*, *Banji long* and *Khaan mckennai* [27, 51, 52] but in contrast to the complete fusion of the premaxillae seen in *Citipati osmolskae* and *Nemegtomaia barsboldi* [53, 54]. The premaxilla contacts the nasal posterodorsally, the lacrimal posteriorly and the maxilla posteroventrally. The height of the subnarial portion of the premaxilla is roughly equal to the anteroposterior length of the premaxillary tomial margin. It should be noted that the general shape of this region varies among oviraptorosaurs both interspecifically and ontogenetically. In basal oviraptorosaurs, the ratio of the anteroposterior length of the premaxillary tomial margin to the dorsoventral height of the premaxillary subnarial region is about 1.0 and 1.7 in *Incisivosaurus gauthieri* and *Caudipteryx zoui*, respectively. In oviraptorids, this ratio is usually 0.7 or less in mature specimens, implying a dorsoventrally high subnarial region. Immature oviraptorid specimens usually have a value similar to that in *Incisivosaurus gauthieri*: for example, IVPP V20182 and the juvenile holotype of *Yulong mini* (HGM 41 HIII-0107) have values of 1.0 and 1.4, respectively ([28]: Table S1). This indicates that the subnarial region of the premaxilla tends to deepen during postnatal development in oviraptorids.

The lateral surface of the subnarial portion of the premaxilla is concave except near the rostral and ventral margins, a condition reminiscent of the subnarial fossa present in *Banji long* [27]. Within this region, a sizeable subtriangular opening lies anteroventral to the external naris (Figs. 5a, b). The corresponding part of the premaxilla bears numerous randomly distributed foramina in *Khaan mckennai*, which probably transmitted small divisions of the medial branch of the ophthalmic nerve (CN V_1_) to innervate the beak [52]. The subtriangular opening is irregular in shape, and may represent the result of breakage of the thin embryonic bone. It should be noted that a similar opening is also present in *Yulong mini*, but is more regular in shape and was interpreted as a diagnostic feature by Lü et al. [28]. The lateral surface of the premaxilla is rugose near the ventral margin, suggesting that a keratinous rhamphotheca may have been present even prior to hatching. Numerous vascular foramina are present in this area, and a row of five large foramina lies roughly parallel with the ventral margin of the beak. The anterior part of the ventral margin of the beak curves medially, so that the triturating surface is U-shaped in ventral view as in other oviraptorosaurs [53, 55].

The nasal process of the premaxilla is a small bony ridge that slants posterodorsally at approximately 45° relative to the horizontal axis of the skull (Figs. 5a, b). The orientation of this process varies interspecifically among adult oviraptorids. In crested taxa such as *Citipati osmolskae* (IGM 100/978), *Nemegtomaia barsboldi* (GIN100/2112) and *Conchoraptor gracilis* (ZPAL MgD-I/95), the nasal process of the premaxilla forms the anterior edge of the protruding crest, and is almost vertically oriented as a result [50, 53, 56, 57]. In *Khaan mckennai* (IGM 100/1127 and 100/1002), by contrast, the crest is minimally developed and the nasal process of the premaxilla is posterodorsally oriented, conforming to the more recumbent profile of the snout [52, 56]. The absence of a crest in immature oviraptorids (e.g. IVPP V20182 and *Yulong mini*, HGM 41HIII-0107) correlates with the posterodorsal orientation of the nasal process of the premaxilla, which indicates that the orientation of the process must have changed during ontogeny at least in crested taxa. It should be noted that the nasal process of the premaxilla is also vertically oriented in the embryonic specimen IGM 100/971 [22]. However, this may represent a preservational artifact, as the skull of this specimen is highly fragmentary.

The subnarial process of the premaxilla is dorsoventrally wider than the nasal process, as in most other oviraptorosaurs including *Yulong mini* [28]. The lateral surface of the subnarial process lacks a depression, as in adults of some oviraptorosaurs such as *Incisivosaurus gauthieri* (IVPP V13326) and *Oviraptor philoceratop*s (AMNH 6517) but in contrast to adults of other oviraptorosaurs including *Khaan mckennai* (IGM 100/1127), *Caudipteryx zoui* (IVPP V12430), *Conchoraptor gracilis* (ZPAL MgD-I/95) and *Citipati osmolskae* (IGM 100/978) [52]. This process contacts the nasal dorsally and the lacrimal body posteriorly, at a position midway down the lacrimal shaft. The subnarial process also slightly overlaps a small portion of the anterior process of the lacrimal, which contributes to the anterodorsal border of the external antorbital fenestra (Figs. 5a, b). In adults of the oviraptorosaurs *Khaan mckennai* (IGM 100/1127), *Conchoraptor gracilis* (ZPAL MgD-I/95), *Citipati osmolskae* (IGM 100/978), *Nemegtomaia barsboldi* (GIN100/2112) and *Incisivosaurus gauthieri* (IVPP V13326), the subnarial process of the premaxilla extends further dorsally along the anterodorsal margin of the lacrimal [50, 55, 58]. In *Yulong mini*, the subnarial process of the premaxilla does not posteriorly contact the lacrimal, representing an autapomorphy of this taxon [28].

The external naris is approximately 6.8 mm long (long axis) and 4.5 mm wide (short axis), giving it an elliptical appearance (Figs. 5a, b). This morphology is similar to that seen in *Yulong mini* (HGM 41HIII-0107), *Khaan mckennai* (IGM 100/1127), *Conchoraptor gracilis* (ZPAL MgD-I/95), *Banji long* (IVPP V16896) and *Wulatelong gobiensis* (IVPP V18409), but different from that seen in *Incisivosaurus gauthieri* (IVPP V13326), *Citipati osmolskae* (IGM 100/978), *Rinchenia mongoliensis* and *Ajancingenia yanshini* [50, 51, 53], in which the external naris is nearly circular. The external naris is roughly as large as the external antorbital fenestra, as in *Khaan mckennai* (IGM 100/1127), *Conchoraptor gracilis* (ZPAL MgD-I/95) and *Citipati osmolskae* (IGM 100/978) but in contrast to *Incisivosaurus gauthieri* (IVPP V13326) and *Caudipteryx zoui* (IVPP V12430), in which the external antorbital fenestra is significantly larger than the external naris [52]. The long axis of the external naris is roughly parallel with the premaxillary nasal process, as in most oviraptorosaurs other than *Citipati osmolskae* (IGM 100/978) and an unnamed oviraptorine specimen from Zamyn Khondt, Mongolia [50, 53]. The external naris is positioned high on the rostrum, and its dorsal margin lies above the level of the dorsal apex of the external antorbital fenestra (Figs. 5a, b) as in all known oviraptorids [59]. The anteroventral corner of the external naris is located at the same dorsoventral level as the dorsal apex of the external antorbital fenestra, as in *Citipati osmolskae* (IGM 100/978), *Khaan mckennai* (IGM 100/1127) and *Huanansaurus ganzhouensis* [60]. In the basal oviraptorosaurs *Incisivosaurus gauthieri* (IVPP V13326) and *Caudipteryx zoui* (IVPP V12430), and in the oviraptorids *Conchoraptor gracilis* (ZPAL MgD-I/95), *Banji long* (IVPP V16896), *Wulatesaurus gobiensis* (IVPP V18409) and *Yulong mini* (HGM 41HIII-0107), the external naris descends to a level well ventral to that of the dorsal apex of the external antorbital fenestra.

The edentulous maxilla is dorsoventrally shallower than in most adult oviraptorids (Figs. 5a, b). The maxilla contacts the premaxilla anteriorly and the lacrimal posterodorsally, and would contact the jugal posteriorly if the jugal were completely preserved. The subnarial process of the premaxilla completely excludes the maxilla from the narial border as in adult oviraptorids [50]. Two small ovoid fossae and a shallow groove are present on the lateral surface of the maxilla along the ventral margin. The jugal process of the maxilla is nearly parallel with the long axis of the skull, forming an acute angle with the descending process of the lacrimal (Figs. 5a, b). This morphology resembles that seen in the basal oviraptorosaur *Incisivosaurus gauthieri* (IVPP V13326) and the juvenile holotype of the oviraptorid *Yulong mini* (HGM 41HIII-0107). In all known mature oviraptorids, however, the jugal process of the maxilla trends posterodorsally and is nearly perpendicular to the descending process of the lacrimal [50, 53, 55]. The jugal process of the maxilla and the lacrimal descending process form the ventral and posterodorsal borders of the external antorbital fenestra, respectively, whereas the subnarial process of the premaxilla and the anterior process of the lacrimal border the external antorbital fenestra anterodorsally (Figs. 5a, b). This configuration is shared with most oviraptorids except *Yulong mini* and *Rinchenia mongoliensis*, in which the subnarial process of the premaxilla forms only part of the anterodorsal margin of the external antorbital fenestra [28, 50]. The triangular antorbital fossa is twice as long anteroposteriorly as it is high dorsoventrally, rather than slightly taller than long as in adult oviraptorids [50]. Little can be said about the morphology of the internal antorbital fenestra, because the margins of this opening have been damaged.

The nasals are completely fused, representing the only fused pair of cranial elements in this specimen, and the compound element is exposed mainly in right lateral and dorsal views (Fig. 5a). This implies that the paired nasals fused at an early ontogenetic stage in oviraptorids, a pattern that has previously been documented only in a few taxa among theropod dinosaurs [61]. The nasal contacts the premaxilla anteriorly, the lacrimal laterally and the frontal posteriorly, and is entirely excluded from the border of the external antorbital fenestra by the premaxilla as in most oviraptorosaurs other than *Yulong mini*. The nasal has a transversely thin premaxillary process that extends anteroventrally to meet the premaxillary nasal process, but the ventrally descending subnarial process of the nasal has been badly damaged (Figs. 5a, b). The dorsal surface of the nasal is penetrated by several large pneumatic openings as in *Yulong mini* (Figs. 5a, b). These openings are likely to represent, in ontogenetically incipient form, the nasal recesses present in adult *Incisivosaurus gauthieri*, *Citipati osmolskae* and *Khaan mckennai* [51–53, 56].

The right frontal is overlapped anteriorly by the nasal in dorsal view, but a fair bit of its posterior part is missing (Figs. 5a, b). The anterior part of the frontal is sharply anteroventrally deflected, and has a rugose surface that underlaps the posterior extremity of the nasal (Figs. 5a, b). The dorsal surface of the frontal posterior to the ventrally deflected portion is gently convex, probably reflecting the curvature of the underlying forebrain, the frontal sinus system, or both. Laterally, the anterior part of the frontal contacts the posterodorsal process of the lacrimal over a short distance, and this contact continues onto the underside of the roof of the orbit as in most adult oviraptorids [52, 53, 55]. The dorsal orbital border lacks a pronounced supraciliary rim of the kind present in *Incisivosaurus gauthieri* and *Khaan mckennai* [52]. The orbital surface of the frontal has been damaged somewhat but clearly lacks pneumatic pockets, as in *Khaan mckennai* but in contrast to the condition in *Citipati osmolskae* [52].

The triradiate lacrimal is exceptionally well preserved. It has a posterodorsal process that wedges in between the frontal and nasal for a short distance, a descending process separating the antorbital fossa from the orbit, and an anterior process that extends along the ventral edge of the subnarial process of the premaxilla (Figs. 5a, b). The posterodorsal process is anteroposteriorly compressed, in contrast to the stout appearance of this process in adult oviraptorids other than *Khaan mckennai* [52]. The splint-like anterior process is roughly as long as the posterodorsal one, which is clearly proportionally longer than in most adult oviraptorids [50, 52, 53]. The anterior process originates from approximately the dorsoventral midpoint of the lacrimal shaft, extending anteriorly as far as the anteroventral corner of the antorbital fossa (Figs. 5a, b). The anterior process is long in the basal oviraptorosaur *Caudipteryx zoui*, but short in all known adult oviraptorids. The presence in an embryonic oviraptorid of a long lacrimal anterior process resembling that of a basal oviraptorosaur suggests that this processs shortened during ontogeny in oviraptorids. The lateral surface of the anterior process is marked by two fossae, one subcircular and the other elongate (Figs. 5a, b). The subcircular fossa is small and located close to the base of the process, resembling the lacrimal recess present in adult oviraptorosaurs. The lacrimal recess takes the form of a single, deep depression in adult specimens of the basal oviraptorosaur *Incisivosaurus gauthieri* and the oviraptorids *Banji long*, *Nemegtomaia barsboldi* and *Khaan mckennai* [51, 52, 55], though it is divided into multiple smaller pockets in *Citipati osmolskae* and *Wulatelong gobiensis* [53, 62]. However, the elongate fossa just ventral to the subcircular one is likely to be a preservational artifact, as this region is not as well preserved as the vicinity of the lacrimal recess.

The descending process of the lacrimal is bowed anteriorly in lateral view, helping to define the circular shape of the orbit. The concave posterior surface is smooth, and lacks the lacrimal foramen that is present in adults of *Incisivosaurus gauthieri*, *Citipati osmolskae*, *Nemegtomaia barsboldi* and *Khaan mckennai*, and probably in the juvenile individuals that represent the only known specimens of *Yulong mini* [28, 51–53]. The descending process is anteroposteriorly compressed as in most oviraptorosaurs [28, 51–53, 62]. Although damaged to some extent, this process forms a blade-like margin that projects laterally beyond the plane defined by the margins of the orbit (Figs. 5a, b), as is characteristic of all known oviraptorids [50]. Some irregular fossae are visible on this damaged margin, indicating that the lacrimal is highly pneumatic as in mature oviraptorids [52, 57].

The jugal is poorly preserved, apart from the bases of the postorbital and maxillary processes (Figs. 5a, b). The maxillary process extends slightly anterodorsally relative to the horizontal axis of the skull, a feature previously known only in mature oviraptorids. The postorbital process of the jugal is nearly perpendicular to the maxillary process, as in most adult oviraptorids. However, the postorbital process is posterodorsally inclined, forming an obtuse angle with the maxillary process, in the basal oviraptorosaurs *Incisivosaurus gauthieri* and *Caudipteryx zoui*, and in the derived taxon *Wulatelong gobiensis* [50, 62].

The edentulous mandible is represented by the incomplete dentary, surangular and articular, along with what may be the prearticular, all of which are from the right side and appear to be approximately in their natural positions (Figs. 5a, b). The mandible measures approximately 39 mm from the anterior end of the dentary to the posteriormost preserved part of the articular, and 15 mm from from the peak of the coronoid eminence to the ventral margin of the mandible, whose position can be estimated based on the preserved ventral edge of the articular (Table 1). The mandible appears shorter, relative to its depth, than in *Incisivosaurus gauthieri*, *Caudipteryx zoui*, caenagnathids and other theropods [51, 52, 63–70]. The dentary is deep and short, and the symphysial region is dorsoventrally deep. The steeply inclined posterodorsal process of the dentary has a gently concave dorsal margin. A large gap exists between the anterior parts of the upper and lower jaws, and seems to result more from the inclination of the posterodorsal process of the dentary than from the concavity of its dorsal margin. The external mandibular fenestra is large and anteriorly positioned as in oviraptorids generally [28, 52, 53].

Two bony splinters situated ventral to the orbit are tentatively identified as pieces of the surangular (Figs. 5a, b). The larger piece is straplike and slightly dorsally overlapped by the jugal, whereas the other lies posterior to the posterodorsal process of the dentary and probably represents the peak of the coronoid eminence (Figs. 5a, b). The orientation of these two fragments suggests that the intact surangular was inclined posteroventrally at a shallow angle posterior to the eminence, but the unsatisfactory preservation of the surangular makes further details impossible to describe.

The triangular bony element in articulation with the quadrate is presumably the articular (Figs. 5a, b). The articular is transversely wide, and its lateral surface is slightly concave and pierced by a small foramen. This foramen, together with the presence of an anterodorsally facing depression anterior to the joint surface for the quadrate, indicates that the articular was not completely ossified at the time of death (Figs. 5a, b). Posteriorly, the articular tapers to a slender retroarticular process as in other oviraptorids, but only the base of this process is preserved. The joint surface for the quadrate is exclusively formed by the articular (Figs. 5a, b), and faces entirely dorsally. This joint surface is positioned slightly above the level of the mandibular symphysis, a feature consistently present in both juvenile and adult oviraptorids [27, 28, 53]. In other oviraptorids, the anterior part of the midline of the ventral surface of the articular contacts the prearticular, whereas the lateral surface of the articular contacts the surangular [52, 53]. The splinter of bone preserved ventral to the articular could be a fragment of either the caudal part of the surangular or the prearticular (Figs 5a, b).

A total of seven vertebrae are preserved in a continuous series in this specimen, and their centra are all separated by visible sutures from the corresponding neural arches (Fig 5c). The anterior two isolated centra represent the penultimate and last sacrals, as they differ from the other five in being associated with sacral ribs (Fig. 5c). The transverse width of the penultimate sacral centrum is approximately three times as great as the dorsoventral height, and the centrum is excavated by a pleurocoel on either side (Fig. 5c). The pleurocoel is small, anteroposteriorly elongate, and located at the center of the lateral surface of the centrum (Fig. 5c). The last sacral centrum is somewhat spool-like, with relatively smooth lateral and ventral surfaces that respectively lack any sign of pleurocoels and any sign of a keel or groove. These centra are not coossified with the corresponding sacral ribs, and also remain separate from each other. The articular surfaces of the last sacral centrum are visible, revealing that this centrum is amphiplatyan as in adult oviraptorids [50].

The remaining five vertebrae continue in an uninterrupted series from the last sacral and represent the proximal caudals Ca1 through Ca5, though the only preserved part of Ca5 is a broken neural arch (Fig. 5c). These vertebrae rapidly decrease in overall size from anterior to posterior, and represent a more extensive sequence of articulated caudals than is preserved in most other oviraptorid embryos [22–24]. Although the centra are badly eroded, the centrum of Ca1 appears spool-like, without any evidence of a ventral keel or groove. The mediolateral width of the centra decreases more rapidly than their length or height (Fig. 5c). The neural arches of these caudal vertebrae are transversely wider than anteroposteriorly long, primarily because of their long transverse processes (Fig. 5c). The distal ends of the transverse processes are subcircular, like those of *Khaan mckennai* [52], and each bears a tiny depression that faces mostly ventrally. Both the pre- and postzygapophyses are well ossified. The prezygapophyses are longer than the postzygapophyses, and extend primarily anteriorly for a distance that is less than half of the length of the preceding centrum (Fig. 5c). This condition is diagnostic of all oviraptorosaurs, but contrasts with the elongated prezygapophyses of most non-oviraptorosaurian theropods [50, 52, 71, 72].

Only the proximal halves of the right metatarsals II-IV are preserved in IVPP V20182 (Fig. 5e). They are proximally unfinished, and metatarsal III narrows only slightly between metatarsals II and IV. Metatarsal II appears to be slightly more slender than metatarsal IV in anterior view, a feature also present in mature oviraptorids such as the holotype of *Wulatelong gobiensis* [62].

### IVPP V20183 (Fig. 6)

**Fig. 6:**
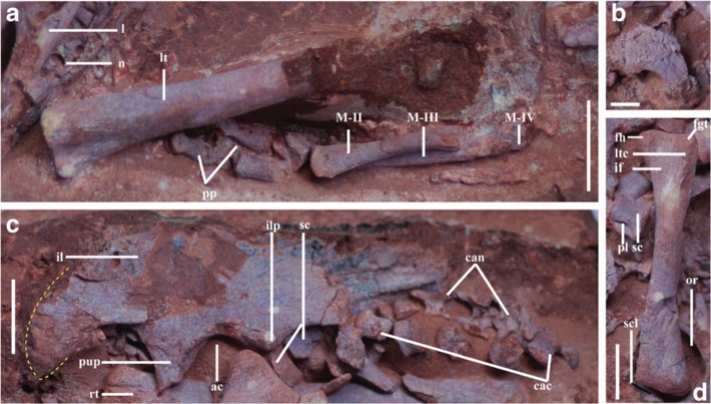
Photographs of IVPP V20183. **a**, magnified view of the left tibia and pedal elements; scale bar = 1 cm; **b**, magnified ventrolateral view of the U-shaped dentary; scale bar = 5 mm; **c**, magnified lateral view of the left ilium and the proximal caudal vertebrae, scale bar = 1 cm; **d**, magnified anterior view of the left femur and the sacral centrum; scale bar = 1 cm. Abbreviations: ac, acetabulum; can, caudal neural arch; cac, caudal centrum; fgt, greater trochanter of femur; fh, femoral head; if, intertrochanteric fossa; il, ilium; ilp, ischial peduncle; l, lacrimal; lt, left tibia; ltc, lesser trochanteric crest; n, nasal; or, orbit; pl, pleurocoel; pp, pedal phalange; pup, pubic peduncle; rt, right tibia; sc, sacral centrum; scl, sclerotic plates; MII-IV, metatarsal II-V. The yellow dotted line demarcates the broken edge of the preacetabular process

#### Histological description

The cross section of the tibia is apparently oval, with a marrow cavity that accounts for a third of the diameter of the section. The cortex is made up of porous fibrolamellar bone that is more compact than that seen in IVPP V20182, with an overall porosity of 20.6 %. The vascular canals range in shape from circular to somewhat elongated along an arc parallel to the circumference of the bone, and the edge of the marrow cavity is well defined (Figs. 8b_1_, b_2_). Most of the osteocyte lacunae are organized around vascular canals, forming incipient primary osteons as in IVPP V20182.

#### Skeletal description

This specimen retains a semiarticulated skeleton that is more complete than those of the other two embryos, preserved in a characteristic fetal position with the head tucked forward next to the knees (Fig. 2b). The specimen is exposed mainly in left lateral view, and preserved elements include the incomplete nasal, lacrimal and frontal, the anterior ends of the dentaries, the proximal caudal vertebrae, the pelvic girdle, and both hindlimbs. The head is located close to one end of the short axis of the egg, with the skull roof appressed against the shell, while the pelvis lies on the opposite side of the egg from the head. The left hindlimb is flexed and remains partially articulated, though the femur has been displaced out of the acetabulum so that its distal end lies in the left orbit (Fig. 6d). The right hindlimb is also flexed, and the tibia is parallel to the long axis of the pelvis as in the Bugin-Tsav embryo MPC-D100/1019-2 [23].

The nasal is incomplete and has been transversely crushed (Fig. 6a). The lacrimal has an elongate anterior process that extends primarily anteroventrally (Fig. 6a). The lateral surface of this process is excavated near the base by an ovoid fossa, which is probably equivalent to the incipient lacrimal recess present in IVPP V20182. Most of the lacrimal descending process is hidden by the femur, although the morphology of the exposed base suggests that the process is similar to the equivalent structure in IVPP V20182 and other oviraptorids.

The frontal is anteroposteriorly short as in all known oviraptorids [50]. The frontal is dorsally convex, and abruptly begins to slope anteroventrally at the point where it presumably began to underlap the nasal in the intact skull. This abrupt slope is also present in IVPP V20182 and *Yulong mini* (HGM 41HIII-0109), but is unlike the gentle curvature present in mature oviraptorids, indicating that this feature changes during ontogeny. The frontal clearly defines the dorsal margin of the orbit, but there is no sign of a supraciliary rim, a feature also absent in IVPP V20182. The orbit is subcircular, and contains some pieces of bone that presumably represent sclerotic plates (Fig. 6d). These plates generally resemble those preserved in MPC-D 100/1019-1 [23] in shape and position, but their detailed morphology cannot be described due to unsatisfactory preservation.

Only the anterior portions of the dentaries are preserved (Fig. 6b), and they articulate loosely at the symphysis as in the Ukhaa Tolgod embryo IGM 100/973 [22]. The dentaries are tightly sutured together in all known post-hatching oviraptorids including the juvenile *Yulong mini*, but are completely fused in *Gigantoraptor erlianensis* and other caenagnathids [52]. The symphysial region is anteroposteriorly extensive, and the two dentaries meet along a straight but jagged suture. The anterior portion of the dentary is downturned in lateral view and U-shaped in ventral view, as in all known oviraptorids [34, 50, 53, 55, 59].

A total of nine unfused vertebrae are preserved in this specimen (Fig. 6c). The three centra lying furthest anteriorly are nearly three times as transversely wide as dorsoventrally high, and bear a large, centrally positioned oval pleurocoel on each side. These features are sufficient to identify them as posterior sacrals, presumably the posteriormost three in the sacral series, but they are scattered near the pelvic girdle so that their order is uncertain. The most posterior sacral centrum is positioned ventral to the postacetabular process of the left ilium, the intermediate one is mostly hidden by the ischial peduncle and the proximal end of the femur (Fig. 6c), and the most anterior one has been displaced to a position adjacent to the pubic peduncle. The centra are amphiplatyan, and their anterior surfaces are transversely wider than their posterior surfaces. The ventral surfaces of the centra are smoothly and gently concave anteroposteriorly. The pleurocoels are much larger and more deeply excavated than those on the penultimate sacral of IVPP V20182. The base of a single rib is preserved in articulation with the most anterior sacral centrum, but the sacral ribs are otherwise missing. Lack of fusion among the sacral vertebrae is a common condition in other immature theropods [18, 73, 74], although at least one oviraptorid embryo has been reported to possess co-ossified sacral centra [22]. The remaining centra are identified as the first six caudals (Fig. 6c). They are spool-like and decrease in size from anterior to posterior, and their flat articular surfaces make them amphiplatyan as in other oviraptorids. It is noteworthy that the number of preserved caudal vertebrae varies systematically in immature oviraptorids at different ontogenetic stages. IVPP V20183 was more ontogenetically advanced than IVPP V20182 at the time of death (see Bone histology and developmental stage estimation, below), and the preserved caudal vertebrae are better ossified and slightly more numerous in the former specimen than in the latter. The juvenile *Yulong mini* (HGM 41HIII-0107) is undoubtedly more advanced than IVPP V20183, and preserves at least 20 proximal caudals [28]. This preservational trend is seemingly indicative of an anterior-to-posterior pattern of caudal vertebral ossification in oviraptorids, but more information will be needed in order to rule out the possibility that it is artifactual.

The pelvic girdle and hindlimbs are normally the best preserved portion of the skeleton in oviraptorid embryos, indicating that they are especially well ossified and/or well held together by ligaments. The pelvis and hindlimbs are best exemplified, among the newly collected embryos, by IVPP V20183 (Figs. 6c, d). The left ilium of this specimen is exposed in lateral view, and is nearly complete except along the dorsal margin and at the anterior and posterior ends. The ilium is moderately deep and dorsally convex, as in other oviraptorids including immature *Yulong mini* [28, 50]. The preacetabular process is longer than the postacetabular process (Table 1), as in adult *Chirostenotes pergracilis*, *Nomingia gobiensis*, *Heyuannia huangi* and *Rinchenia mongoliensis*, and in the embryonic specimen MPC-D100/1019-2 [23, 50, 52, 59]. In *Shixinggia oblita* and *Avimimus portentosus*, however, the postacetabular process is longer than the preacetabular process [50, 75]. *Khaan mckennai* is distinctive in that the postacetabular process is nearly, but not quite, as long as the preacetabular process [52]. In IVPP V20183, the preacetabular process is directed anteroventrally as in other oviraptorosaurs (Fig. 6c), but does not extend as far ventrally beyond the pubic peduncle as in *Rinchenia mongoliensis* and *Caudipteryx zoui* [50, 52, 68]. The pubic peduncle is much broader anteroposteriorly, and extends much further ventrally, than the ischial peduncle (Fig. 6c). A pubic peduncle that extends ventrally beyond the ischial peduncle is present in several adult oviraptorosaurs, including the basal taxa *Caudipteryx zoui* and *Nankangia jiangxiensis*, the putative oviraptorid *Luoyanggia liudianensis*, and the oviraptorid *Wulatelong gobiensis* [33, 54, 62, 68, 69]. In most other oviraptorids, the pubic and ischial peduncles are about equal in ventral extent [50]. *Luoyanggia liudianensis* was assigned to Oviraptoridae in the course of a brief description [54], but appears to lack preserved synapomorphic features that would support such a placement, leaving *Wulatelong gobiensis* as the only definitive oviraptorid with a highly pendant pubic peduncle [62]. IVPP V20183 and MPC-D100/1019-1 (Weishampel, personal communication) are similar to basal oviraptorosaurs and *Wulatelong gobiensis* in having pubic peduncles that extend further ventrally than the ischial peduncles, indicating that the pubic peduncle may have been dorsoventrally elongated in oviraptorid embryos but subsequently have grown shorter in proportional terms after hatching. A cuppedicus fossa is evident when the ilium is examined in lateroventral view, but there is no sign of a brevis fossa, suggesting the latter structure would have formed late in ontogeny. The dorsal margin of the acetabulum has been slightly damaged, but the supraacetabular rim is apparently poorly developed as in *Khaan mckennai* [52].

The paired bones that underlie the left femur are tentatively identified as the pubes, based on their position and weakly anteriorly concave shafts (Fig. 2b). Their proximal ends are hidden by the femur, whereas their distal ends have been broken off. A seriously eroded element that proximally contacts the femur appears to represent the left ischium (Fig. 2b). The ischial shaft is transversely compressed, and its proximal end is anteroposteriorly expanded to form a flange that presumably represents the obturator process.

The exquisitely preserved left femur is nearly straight in lateral view, but slightly sigmoidally curved when viewed posteriorly (Fig. 6d). The femur is approximately as long as the ilium, as in most oviraptorids. In *Rinchenia mongoliensis* (MPC-D100/32a) and *Yulong mini* (HGM 41HIII-0107), however, the femur is significantly longer than the ilium [28, 50]. The proximal end of the femur is not fully ossified, as indicated by the weakly developed greater trochanteric crest and the absence of a constricted femoral neck between the greater trochanter and the femoral head (Fig. 6d). A distinct femoral neck is present in adult *Khaan mckennai* and *Conchoraptor gracilis*, whereas no or little constriction is present in adult *Citipati osmolskae* or *Gigantoraptor erlianensis*, or in the Bugin-Tsav embryonic specimens [23, 52]. The femoral head extends straight medially as is characteristic of all coelurosaurs, and reaches nearly the same proximal level as the greater trochanter (Fig. 6d). The lesser trochanter is a pronounced crest that extends along the anterolateral margin of the femur, though the proximal end of the crest is damaged and poorly exposed (Fig. 6d). This crest angles medially as it extends distally, and has a proximodistal length of approximately 11 mm. Before merging with the anterior surface of the shaft, it thickens distally into a robust protuberance which is likely homologous to the accessory trochanter present in taxa such as *Microvenator celer* [76]. Medial to the lesser trochanter, on the anterior surface of the shaft, is the rugose, gently concave intertrochanteric fossa (Fig. 6d). The shaft of the femur is relatively thin at the midpoint of its length, and nearly circular in cross section. The anterior and lateral surfaces of the shaft are generally smooth at this point, but some longitudinal striations are present on the anterior surface close to the distal condyles, and probably represent the attachment sites of M. femorotibialis externus et internus [77]. The distal articular surface of the femur is poorly defined, with little differentiation into medial and lateral condyles, accentuating the incomplete ossification of the bone.

The right tibia is nearly completely preserved, though it is hidden by several bones including the left femur and ischium (Fig. 2b). The tibia is approximately 114 % as long as the femur, and the equivalent ratio is similar in most oviraptorids including juvenile specimens of *Yulong mini* [28, 50, 52, 59, 62, 69, 78]. However, the tibia is 40 % longer than the femur in MPC-D100/1018 and MPC-D100-1019-2, which were recovered from a single nest [23], probably owing to interspecific variation. The cnemial and fibular crests are only partly ossified, but are clearly separated from each other. The tibia is nearly circular in cross section at a level immediately ventral to the crests but becomes anteroposteriorly flattened more distally, having an elliptical cross section at the midpoint (Figs. 8b_1_, b_2_). Preserved distal hindlimb elements include the incomplete left metatarsals II and III and their corresponding phalangeal series, but these bones are not morphologically informative.

### IVPP V20184 (Fig. 7)

**Fig. 7:**
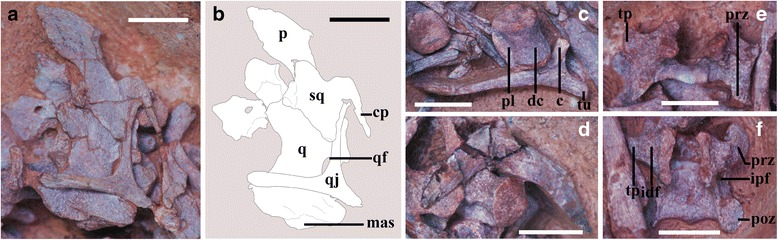
Photographs of IVPP V20184. **a**, magnified left lateral view of the cranial elements; scale bar = 5 mm; **b**, schematic drawing of the cranial elements, scale bar = 5 mm; **c**, magnified view of a dorsal centrum and a dorsal rib; scale bar = 5 mm; **d**, magnified view of the furcula; scale bar = 5 mm; **e**, magnified ventral view of an isolated anterior cervical neural arch; scale bar = 5 mm; **f**, magnified view of an isolated anterior dorsal neural arch; scale bar = 5 mm. Abbreviations: c, capitulum; cp, squamosal caudal process; dc, dorsal centrum; f, furcula; idf, infradiapophyseal fossa; il, ilium; ipf, infrapostzygapophyseal fossa; mas, mandibular articular surface; p, parietal; pl, pleurocoel; poz, postzygapophysis; prz, prezygapophysis; q, quadrate; qf, quadrate foramen; qj, quadratojugal; sq, squamosal; tp, transverse process; tu, tuberculum

#### Skeletal description

The skeleton of IVPP V20184 is disarticulated, and piled up at one end of the egg (Fig. 2c). The left parietal, quadrate, squamosal and quadratojugal are preserved, along with a few cervical and dorsal vertebrae, some dorsal ribs, the nearly complete furcula and a possible femur, but most of the skeleton has been lost to weathering.

The parietal is represented only by a fragment of bone that lies dorsal to the squamosal (Figs. 7a, b). The squamosal is laterally concave (Fig. 7a), with a small posteroventrally projecting posterior process and a ventrally projecting quadratojugal process. The quadratojugal process contacts the squamosal process of the quadratojugal along its posterior margin, and slightly overlaps the latter bone as in *Citipati osmolskae*. The postorbital process of the squamosal is missing.

**Fig. 8:**
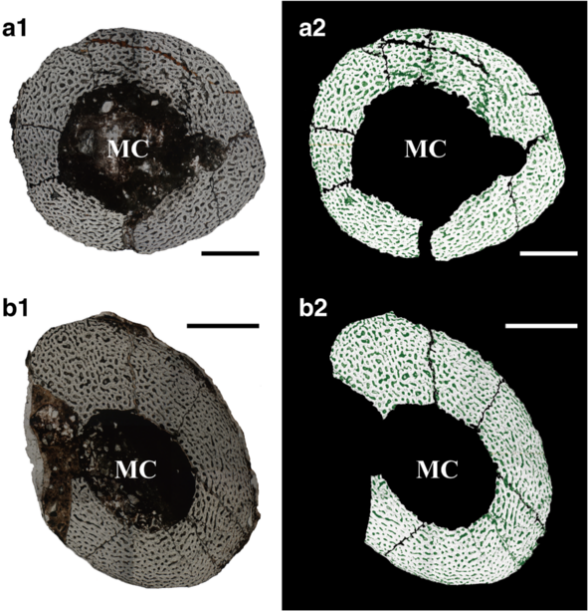
Histological sections of the embryonic skeletons. **a1**, cross section of the tibia of IVPP V20182, showing fibrolamellar bone with relatively large and rounded vascular canals and poorly defined marrow cavity; scale bar = 1 mm; **a2**, the same cross section in **a1** with the vascular canals highlighted in green; scale bar = 1.2 mm; **b1**, cross section of the tibia of IVPP V20183, showing fibrolamellar bone with compacted, longitudinal and concentrically arranged vascular canals, and a well defined marrow cavity; scale bar = 1.2 mm; **b2**, the same cross section in b1 with the vascular canals highlighted in green; scale bar = 1.2 mm; Abbreviations: MC, marrow cavity

The triradiate quadratojugal is complete and exceptionally well preserved, and borders the infratemporal fenestra posteroventrally as in other oviraptorids (Figs. 7a, b). It has a squamosal process that forms an angle of approximately 90° with the jugal process, as in most oviraptorosaurs except adult *Citipati osmolskae* and the embryonic specimen MPC-D 100/1019-1. In *Citipati osmolskae*, the jugal process of the quadratojugal projects anterodorsally, and forms an acute angle with the squamosal process [50, 53]. In MPC-D 100/1019-1, the squamosal and jugal processes of the quadratojugal form an angle of 100°, probably as a result of slight distortion ([23]: Fig. 8). The squamosal process extends dorsally to the base of the posterior process of the squamosal (Figs. 7a, b). Although the squamosal-quadratojugal articulation is somewhat distorted, the squamosal process of the quadratojugal borders most of the posterior edge of the infratemporal fenestra as in other oviraptorids [50]. The jugal process is roughly as long as the squamosal process, and terminates in an anteriorly tapering, laterally facing articular surface that would have contacted the jugal (Fig. 7a). The posterior process extends primarily posteriorly and only slightly ventrally, closely resembling the equivalent structure in adult *Conchoraptor gracilis* [50]. This process is posteroventrally directed in adult *Citipati osmolskae* and *Rinchenia mongoliensis*, and in the unnamed adult Zamyn Khondt oviraptorine [50, 52, 53], but projects straight ventrally in the basal oviraptorosaur *Incisivosaurus gauthieri* [51]. In *Khaan mckennai* the posterior process projects posteroventrally, but is greatly reduced relative to that of *Citipati osmolskae* [52].

The left quadrate is well preserved and exposed in both ventral and lateral views (Figs. 7a, b). The quadrate is massive and lacks the pneumaticity that characterizes this bone in adult oviraptorids [50–52, 57, 79], tyrannosaurids [71, 80–83], troodontids [84, 85], and basal avialans [86]. The quadrate has a thin but greatly expanded pterygoid process that extends primarily anteriorly. The ventral margin of this process has a complex curvature, extending ventrally beyond the level of the glenoid surface and merging posteriorly with the medial condyle, as is characteristic of all oviraptorids (Figs. 7a, b) [79]. Dorsal to the mandibular articular surface, on the lateral surface of the quadrate, is a concave facet for the quadratojugal, though there is no sign of a quadrate lateral process. Maryańska & Osmólska pointed out that the presence of a lateral process on the mandibular condyle and a concave facet for the quadratojugal distinguishes the oviraptorid quadrate from those of other non-avian theropods [79]. However, this combination of features is not always present even in adult oviraptorids. In *Nemegtomaia barsboldi*, for example, the lateral process is absent and the articular surface for the quadratojugal is convex rather than concave [55]. In *Heyuannia huangi*, the lateral process is also absent, but the articular surface for the quadratojugal is concave [59], reminiscent of the comdition in IVPP V20184. The posterior surface of the quadrate is concave as in other non-avian theropods and *Archaeopteryx* [79, 87], and this surface also forms the medial margin of the quadrate foramen (Figs. 6a, b). However, little can be said about the morphology of the quadrate foramen because the quadratojugal has been displaced relative to the quadrate.

The mandibular process of the quadrate is mediolaterally wide. The mandibular condyles are indistinct due to poor ossification, but are separated ventrally by a wide and shallow anteroposteriorly trending intercondylar depression. The medial condyle extends slightly beyond the lateral one both anteriorly and ventrally. The articular surfaces of both condyles are more convex anteroposteriorly than mediolaterally, and are only weakly inclined towards the intercondylar depression. In ventral view, the lateral condyle has a subtriangular appearance, whereas the medial condyle is somewhat wider than the lateral condyle and is roughly rectangular in shape. This morphology is reminiscent of the condition in *Citipati osmolskae*, but contrasts with that in *Nemegtomaia barsboldi*, in which the medial condyle is oval in ventral view and the lateral one is strap-like.

The vertebrae preserved in IVPP V20184 are scattered throughout the region of the egg pole, and the centra are separated by visible sutures from their neural arches as in all known embryonic oviraptorids (Fig. 2c). Only a single centrum and two neural arches, positioned just posterior to the cranial elements, can be identified with certainty as part of the cervical series. The anteroposterior length of the centrum (not shown) is twice as great as the dorsoventral depth, and the centrum is excavated by a small circular pleurocoel situated nearly in the center of the lateral surface. The presence of a single pleurocoel on each lateral surface of each cervical centrum is characteristic of nearly all oviraptorosaurs in addition to many other theropods, although the oviraptorosaur *Microvenator celer* possesses two pleurocoel pairs per centrum [52, 76]. In adult oviraptorids the pleurocoels are anteriorly located on the anterior cervicals and progressively shift toward the center of the lateral face in more posterior ones [52], which implies the isolated centrum of IVPP V20184 probably comes from the dorsocervical region. Anterior to the pleurocoel, on the anteroventral corner of the centrum, is a large, ovoid parapophysis. The parapophysis protrudes laterally from the centrum only to a small degree, whereas in adult oviraptorids the cervical parapophyses are set off on ventrolaterally projecting pedicles [50].

The detached neural arch located posterior to the cranial elements is missing the posterior half and exposed mainly in ventral view (Fig. 7e). The prezygapophyses are short, anteriorly directed, and roughly parallel to each other. Their articular surfaces face medially as much as dorsally. Each transverse process appears triangular in ventral view (Fig. 7e), and is roughly horizontally directed with a slight posterior curvature. The articular surface for the tuberculum of the rib is unfinished, but ventrally projecting anterior and posterior centrodiapophyseal laminae are incipiently developed, defining a shallow infradiapophyseal fossa that faces equally ventrally and laterally. Another isolated neural arch lies close to the egg pole (Fig. 7f), and possesses anterodorsally projecting prezygapophyses that diverge more strongly from the midline than in the previous neural arch. The articular surfaces of the prezygapophyses are not well exposed, but can be inferred from the orientation of the prezygapophyseal bases to face primarily dorsally and slightly medially. The articular surfaces of the postzygapophyses are oval, and face mostly ventrally and only slightly laterally (Fig. 7f). The postzygapophyses are connected by an osseous lamina, which forms a slight posterior prominence at its midpoint (Fig. 7f). The infrapostzygapophyseal fossa is larger and more deeply excavated than the infradiapophyseal fossa (Fig. 7f), and is well exposed in both ventral and posterior views.

Although their exact positions in the column are difficult to determine, the remainder of the scattered centra may come from dorsal vertebrae. This inference is supported by a combination of features including the spool-like appearance of the centra, the presence of anteroposteriorly elongate pleurocoels on their lateral faces, and the lack of parapophyses (Fig. 7c). The articular surfaces of the centra are flat to minimally concave. The elongate pleurocoels are larger and deeper than those on the cervicals. The ventral surfaces of these centra are rounded and smooth, without any sign of ventral keels or grooves. In adults of the oviraptorid *Khaan mckennai*, hypapophyses are present in the dorsal series, but are restricted to the anteriormost three to four dorsal vertebrae [52]. A few fragmentary dorsal ribs are preserved in association with the dorsal centra, with capitula and tubercula that are well separated from each other (Fig. 7c). Other features of the ribs are difficult to observe, owing to the poor ossification of these bones.

The furcula is well preserved and exposed in anterior view (Fig. 7d). The interclavicular angle is about 100°, slightly greater than the 90° angle reported in adult *Oviraptor philoceratops*, *Khaan mckennai* and *Heyuannia huangi* [52, 88]. There is no sign of an interclavicular suture (the fissure visible in Fig. 7f is an artifact created during preparation), as is also the case in the Ukhaa Tolgod oviraptorid embryo IGM 100/971 [22]. The furcula is thickest in the area of symphysial fusion, and each lateral process gradually becomes anteroposteriorly compressed as it extends distally. In anterior view, each lateral process is sharply kinked at a point approximately 60 % along its length, and tapers distally beyond this point to form an epicleidium with a slight medial incurving.

## Discussion

### Taxonomic identification of the embryonic skeletons

The newly collected embryonic specimens can be unambiguously identified as oviraptorosaurs, and more specifically as oviraptorids, based on a range of well-established diagnostic features [50]. Oviraptorosaur synapomorphies visible in the specimens include: crenulated ventral margin on premaxilla (IVPP V20182); squamosal process of quadratojugal borders more than three-quarters of posterior edge of infratemporal fenestra (IVPP V20184); U-shaped mandibular symphysis (IVPP V20183); and caudal vertebrae with short and anterodorsally directed prezygapophyses (IVPP V20182 and IVPP V20183). Visible oviraptorid synapomorphies include: short snout (IVPP V20182); pneumatized premaxilla (IVPP V20182); subantorbital portion of maxilla inset medially (IVPP V20182); external naris extends ventrally beyond level of dorsal margin of external antorbital fenestra (IVPP V20182); anteroposteriorly compressed and laterally protruding descending process of lacrimal (IVPP V20182); pneumatized nasal and frontal (IVPP V20182 and IVPP V20183); concave quadratojugal facet on quadrate (IVPP V20184); deep mandible (IVPP V20182); and toothless premaxilla (IVPP V20182) [50].

Six oviraptorid taxa have been previously reported from a small geographic area straddling the border between Guangdong and Jiangxi Provinces in southern China: *Banji long* [27], *Ganzhousaurus nankangensis* [34], *Jiangxisaurus ganzhouensis* [35], *Heyuannia huangi* [59], *Shixinggia oblita* [75], and *Huanansaurus ganzhouensis* [60]. All of these species are represented only by their holotypes, which are considered to have been adult individuals with the exception of the juvenile holotype of *Banji long* [27, 34, 35, 59, 75]. All of them have features that seem to distinguish them from the new embryonic individuals described above. *Banji long* has an elongate external naris and bears longitudinal grooves on both the moderately developed crest and the posterior part of the dorsal margin of the dentary [27]; *Jiangxisaurus ganzhouensis* has an elongate mandible, characterized by a ratio of maximum height to maximum length of only about 20 % [35]. In *Heyuannia huangi* the anterior end of the dentary is exceptionally elongate and strongly downturned [59]. In *Shixinggia oblita* the ventral margins of the pre- and postacetabular processes are situated above the level of the dorsal margin of the acetabulum, and the preacetabular process is shorter than the postacetabular process [75]. In *Huanansaurus ganzhouensis*, the squamosal process of the quadratojugal forms an angle of approximately 65° with the jugal process [60]. Although none of these features is present in any of the newly collected specimens, the possibility that one or more of them may have emerged in the course of post-hatching development cannot be ruled out. Comparisons between the new specimens and *Ganzhousaurus nankangensis* are impossible, because the only elements preserved in the single known individual of this species (SDM 20090302) are absent in the new embryos.

The only non-oviraptorid oviraptorosaur known from this area is the recently described *Nankangia jiangxiensis*, which differs from all known oviraptorids including IVPP V20182 and V20183 in having a dentary with a gently inclined posterodorsal process but without a downturned anterior end [33]. This species in fact resembles IVPP V20183 in possessing a pubic peduncle that is deeper than the ischial peduncle [33]. However, this feature cannot be taken as a strong indication that IVPP V20183 might be referable to *Nankangia jiangxiensis*, because it is not autapomorphic. A ventrally pendant pubic peduncle has also been documented in adult specimens of *Caudipteryx zoui*, *Luoyanggia liudianensis* and *Wulatelong gobiensis* [54, 62, 69], probably representing a plesiomorphic feature for oviraptorosaurs.

Considering that the newly collected embryos lack any evident autapomorphic features that could be used to erect new species, we prefer to consider them as Oviraptoridae *incertae sedis*, pending the recovery of new information. Additional specimens from the Guangdong-Jiangxi border region might eventually demonstrate that all three of the embryos described in this paper are referable to one or more previously described species, or might provide strong justification for recognizing at least one of them as a member of a new species.

### Bone histology and developmental stage estimation

The tibial thin sections from IVPP V20182 and IVPP V20183 show typical reticular tissue, characterized by fibrolamellar bone with high porosity (Fig. 7) [40]. A poorly-defined marrow cavity is the direct result of rapid deposition of bone, resorption of endosteal tissue, and shifting of the marrow cavity over time; whereas a well-defined marrow cavity is usually indicative of a relatively low rate of deposition [89]. The tibial section of IVPP V20183 has a relatively smaller and better defined marrow cavity than that of IVPP V20182. This condition, the relatively small, longitudinal and concentrically arranged vascular canals, and the relatively low overall amount of vascularization (Fig. 8), all indicate that IVPP V20183 was growing more slowly than IVPP V20182, perhaps indicating that the former specimen was more ontogenetically advanced at the time of death. IVPP V20183 is also slightly larger than IVPP V20182, based on a comparison of tibial diameter between the two specimens (Table 1). Neither IVPP V20182 nor IVPP V20183 exhibits any signs of osseous pathology. The ontogenetic status of IVPP V20184 relative to the other specimens is difficult to determine because no tibia is available for sectioning.

### Anomalous eggshell features

In all three new specimens, double-layered and multilayered cones are present, protruding into the columnar layer of the shell (Fig. 4b). The extra cones are usually present above troughs in the boundary between the normal cone and columnar layers and beneath peaks in the outer surface of the eggshell, forming an extra layer that is half as thick as the normal cone layer. Extra cones of this type have been widely reported in elongatoolithid and macroelongatoolithid eggshells, including specimens from the Nanxiong Basin in Guangdong Province, China [42–45], the Tiantai Basin in Zhejiang Province, China [90], Emery County in Utah, United States of America and Aphae-do in Jeollanman-do Province, South Korea [91]. Previous studies have suggested that eggshells with extra cones collected from the Nanxiong Basin came from strata that also contained anomalously high concentrations of environmental trace elements such as Ir, Cr and Zn [42–45, 92]. An experiment in which living female ostriches and chickens were exposed to high Ir levels through food, water and air resulted in Ir deposition in the shells of their eggs [90]. Although the shells were not examined for anomalous microstructural patterns, it was suggested that trace element intake by female dinosaurs might have produced the anomalous shell structures seen in the fossil eggs from the Nanxiong Basin [92]. Assimilated trace elements may have damaged the reproductive systems of the dinosaurs in ways that affected the protein structure of the organic matrix produced during eggshell formation, eventually leading to the observed microstructural abnormalities [43–45, 92]. The extra cones would have thickened the eggshell and blocked some of the pores, and might have had a detrimental effect on hatchability [43, 44]. However, the biochemical mechanism that might cause high trace element levels within an eggshell to produce extra cones remains elusive.

Given the fact that Ganzhou is geographically very close to the Nanxiong Basin, the pathological dinosaur eggshells associated with the newly collected embryos might have formed through the same mechanism, though this supposition remains to be proven. To our knowledge, there is no documented diagenetic mechanism capable of producing extra cone layers in an avian or non-avian dinosaur egg that was originally normal in structure, so diagenetic effects do not represent a likely alternative explanation for the anomalous features observed in the eggshells. However, there are no anatomical or histological indications of pathology in the skeletons of the newly collected embryos, despite the presence of pathological structures in the eggshells. This suggests that trace elements may have affected eggshell formation, but not skeletal development, in this particular case.

### Ontogenetic implications

Ontogenetically variable characters are usually detected by documenting morphological differences between immature and mature individuals of a particular species. However, general ontogenetic variations that apply within a more inclusive clade can sometimes be discovered through comparisons among specimens of different species belonging to the clade. Based on currently available specimens, we identify 20 features that undergo modification over the course of development in oviraptorids (Table 3). It is particularly notable that some features regarded as oviraptorid synapomorphies, such as the dorsoventrally deep subnarial portion of the premaxilla, the anteroventrally sloping ventral margin of the maxilla, and the pneumatic quadrate, appear only late in ontogeny. This implies that very immature specimens may be difficult to confidently assign to Oviraptoridae even when they do belong to this clade, because they will lack some characteristic oviraptorid features. In oviraptorid evolution, a number of derived traits can be inferred to have appeared through modifications of the late steps in development.

**Table 3:** Characters that change during ontogeny in the Oviraptoridae

Feature	State (0): Immature	State (1): Mature
1. Subnarial portion of premaxilla	Shallow	Deep
2. Inclination of nasal process of premaxilla relative to horizontal axis of skull	Approximately 45°	More than 45°
3. Lateral surface of premaxillary body	Bears a single fenestra	Bears a group of foramina
4. Maxilla	Shallow	Deep
5. Maxillary ventral margin	Horizontal	Anteroventrally inclined
6. External antorbital fenestra	Shallow	Deep
7. Angle between jugal process of maxilla and descending process of lacrimal	Less than 90°	Roughly 90°
8. Anteroventral deflection of anterior part of frontal	Abrupt	Smooth
9. Lacrimal posterodorsal process	Anteroposteriorly compressed	Stout
10. Lacrimal anterior process	Long	Short
11. Crest	Absent	Moderately or well developed
12. Pneumatic quadrate	Absent	Present
13. Neural arches and centra	Unfused	Fused
14. Parapophyses on anterior cervicals	Protrude only slightly ventrolaterally	Extend far laterally
15. Sacral centra	Dorsoventrally compressed	Spool-like
16. Distal caudal vertebrae	Unossified	Ossified
17. Furcula hypocleidium	Absent	Present
18. Pubic peduncle	Extends further ventrally than ischial peduncle	Extends as far ventrally as ischial peduncle
19. Brevis fossa	Absent	Present
20. Femoral neck	Absent	Present

Studies of ontogenetic development of theropod dinosaurs are limited by the lack of specimens representing very early ontogenetic stages for most clades [6]. To date, the most thorough investigation of ontogenetic differences between embryonic and more mature theropods was based on therizinosauroid material [20]. However, previous studies have demonstrated that most coelurosaurs probably retained a cranial growth trajectory similar to the plesiomorphic tetanuran one [20, 74, 93], which is dominated by anteroposterior elongation of the craniofacial elements during ontogeny [6, 94]. Such a cranial growth trajectory would result in elongation of the premaxilla, maxilla and external antorbital fenestra. In oviraptorids, however, a series of ontogenetic changes, including deepening of the subnarial portion of the premaxilla, dorsoventral deepening of the maxilla and external antorbital fenestra, anteroventral inclination of the maxillary ventral margin, thickening of the lacrimal posterodorsal process, and shortening of the lacrimal anterior process, collectively cause the dorsoventral height of the skull to increase more rapidly than the anteroposterior length. Among other theropods, a similar ontogenetic pattern is seen only in derived tyrannosaurids [10, 12], in which it presumably appeared independently.

Fusion of the nasal bones across the midline is another point of similarity between oviraptorids and tyrannosaurids. Our results indicate that the nasals of oviraptorids fused in the late stages of embryonic development, and nasal fusion has also been documented in juvenile tyrannosaurids [61]. However, nasal fusion may have differed in functional significance, at least to a degree, between tyrannosaurids and oviraptorids. In tyrannosaurids, nasal fusion appears to have made the snout more resistant to vertical shearing forces produced by unilateral biting [95]. In oviraptorids, nasal fusion may also have contributed to strengthening the skull, but also facilitated the growth of a midline crest that probably served at least partly as a display structure. However, it seems possible that oviraptorids underwent a dietary shift during ontogeny as the skull became allometrically taller during growth, as has previously been proposed for tyrannosaurids [95]. Particularly given that oviraptorids are thought to have been strict herbivores, this would represent an example of parallel evolution of an ontogenetic pattern linked to a shift in feeding behavior.

In modern birds, there are few consistent differences between precocial and altricial species in either the pattern of ossification during embryonic growth or the degree of skeletal development at the time of hatching [96]. However, the observed separation throughout the vertebral column between the neural arches and corresponding centra, and between the vertebrae and corresponding ribs, suggests that the oviraptorid embryos may not share the skeletal ossification pattern previously documented for therizinosauroids, in which the cervical centra, neural arches and even ribs all reportedly fuse during embryonic development [20]. However, ontogenetic stages are difficult to compare precisely between the two clades. If the newly collected oviraptorid embryos were ontogenetically less advanced than the therizinosauroid embryos described by Kundrát et al. [20] at the time of death, then fusion of the neurocentral sutures might have occurred in the subsequent embryonic development of the oviraptorid embryos, had they survived until hatching. If the newly collected oviraptorid embryos were at least as ontogenetically advanced as the therizinosauroid embryos at the time of death, however, the lack of neurocentral fusion in the former would represent a genuine difference in the pattern of embryonic ossification between oviraptorids and therizinosauroids.

The oviraptorid furcula is unique among those of non-avian theropods in being a robust structure with a distinct hypocleidium [48, 88]. In modern birds, the furcula initially ossifies from a bilateral pair of separate centers, and there is no sign of an additional center for the hypocleidium [97]. Instead, ossification continues ventrally, forming a hypocleidium, after reaching the midline. However, the hypocleidium is absent in all reported oviraptorid embryos, including those described in the present study, probably indicating that this structure appeared late in oviraptorid development. Interestingly, a well developed hypocleidium is present in embryonic therizinosauroids but absent in adult specimens [20], implying that the developmental trajectory of this structure in therizinosauroids may be the reverse of that seen in oviraptorids.

Measurements of juvenile specimens of the oviraptorid *Yulong mini* ([28]: supplementary data) indicate that the forelimb length (humerus + ulna + MC II) is less than two thirds of the hindlimb length (femur + tibia + MT III), as in adult oviraptorids. This condition may have characterized oviraptorids throughout their ontogeny, although further examples of juveniles with intact forelimbs and hindlimbs are needed to confirm this. In embryonic specimens that preserve both the humerus and the femur, the length ratio between these two bones is less than 60 % [23], further suggesting that the hindlimb would have been considerably longer than the forelimb in a complete individual. In therizinosauroid embryos, by contrast, the forelimbs are almost as long as the hindlimbs [20]. This not only represents an additional difference in growth trajectory between oviraptorids and therizinosauroids, but also correlates with behavioral variations between the hatchlings of the two groups. Both oviraptorids and therizinosauroids are bipedal as adults, but therizinosauroid hatchlings have been interpreted as quadrupeds based on their limb proportions [20]. The short humeri of oviraptorid embryos, however, suggest that hatchlings were probably bipedal in this clade. Although oviraptorids and therizinosauroids occupy broadly the same grade of maniraptoran evolution, their growth trajectories, locomotor modes and even lifestyles during early post-hatching ontogeny were seemingly very different.

In therizinosauroids, the forelimb may have been negatively allometric relative to the hindlimb during ontogeny, but the hindlimb segments appear to have been nearly isometric with respect to one another [20, 98]. However, differences in hindlimb proportions exist among embryonic oviraptorids. The length ratio of the tibia to the femur is around 1.4 in the Mongolian specimens MPC-D100/1018, MPC-D100/1019-2 and IGM 100/971 [23], but only 1.1 in the southern Chinese specimen IVPP V20183. Considering the fact that the tibia is 7 ~ 27 % longer than the femur in all known post-hatching oviraptorosaur specimens in which the ratio can be measured, including the giant taxon *Gigantoraptor erlianensis* and immature specimens of *Yulong mini* [28, 50, 99], these results probably suggest that growth of the hindlimb was approximately isometric in southern Chinese oviraptorids but characterized by negative allometry of the tibia relative to the femur in at least some Mongolian ones. However, there is no solid evidence for taxonomic separation between the Mongolian and southern Chinese specimens.

## Conclusion

Three newly collected elongatoolithid eggs from Nankang District, Ganzhou City, Jiangxi Province, southern China, each containing a relatively complete *in ovo* oviraptorid dinosaur skeleton, are reported in this paper. The specimens display pathological eggshell structures, but the skeletons of the preserved embryos are structurally and histologically normal, which suggests the environmental factor that led to pathology in the eggshells might not have affected the skeletal development of the embryos. Comparisons between the new embryos and other oviraptorid specimens reveal 20 osteological features that appear to change substantially during ontogeny in oviraptorids. Many of these ontogenetically variable features contribute to dorsoventral deepening of the skull during ontogeny, and early fusion of the nasals may facilitate the later growth of a crest. Although oviraptorids and therizinosauroids occupy broadly the same grade of maniraptoran evolution, the embryonic ossification patterns of the vertebral column and furcular hypocleidium appear to differ significantly between the two clades. The limb proportions of juvenile oviraptorids indicate that they were bipedal, like adults. Oviraptorids may have differed greatly from therizinosauroids in their growth trajectories and locomotor modes during early post-hatching ontogeny, essentially occupying a different ecological niche.

### Availability of data and materials

No additional data are associated with this paper.
